# Benefits of successful percutaneous coronary intervention in chronic total occlusion patients with diabetes

**DOI:** 10.1186/s12933-022-01708-0

**Published:** 2022-12-05

**Authors:** Shuai Zhao, Yan Chen, Qingyi Wang, Boda Zhu, Zhihong Wei, Ziwei Wang, Jiayi Wang, Yiming Zou, Wentao Hu, Cheng Liu, Tiantong Yu, Peng Han, Li Yang, Huan Wang, Chenhai Xia, Qiling Liu, Wei Wang, Haokao Gao, Chengxiang Li, Kun Lian

**Affiliations:** 1 Department of Cardiology, Xijing Hospital, The Fourth Military Medical University, 169 West Changle Road, Xi’an, 710032 Shaanxi People’s Republic of China; 2 Department of Cardiology, No.971 Hospital of the PLA Navy, Qingdao, 266071 Shandong People’s Republic of China; 3 Department of Foreign Languages, School of Basic Medicine, The Fourth Military Medical University, Xi’an, 710032 Shaanxi People’s Republic of China; 4 Cadet Brigade, School of Basic Medicine, The Fourth Military Medical University, Xi’an, 710032 Shaanxi People’s Republic of China; 5 Department of Cardiology, 981 Hospital of Joint Logistics Support Force, Chengde, 067000 Hebei People’s Republic of China; 6 Department of Epidemiology and Medical Statistical, School of Public Health, Shaanxi University of Chinese Medicine, Xianyang, 712046 Shaanxi People’s Republic of China; 7 Department of Pharmaceutics and Pharmacy Administration, School of Pharmacy, The Fourth Military Medical University, Xi’an, 710032 Shaanxi People’s Republic of China; 8 Primary Flight Training Base, Air Force Aviation University, Harbin, 150100 Hei Longjiang People’s Republic of China

**Keywords:** Chronic total occlusion, Diabetes, Clinical outcomes, Symptoms, Quality of life

## Abstract

**Background:**

Diabetes was commonly seen in chronic total occlusion (CTO) patients but data regarding the impact of successful percutaneous coronary intervention (PCI) on clinical outcome of CTO patients with diabetes was controversial. And importantly, no studies have compared quality of life (QOL) after CTO-PCI in patients with and without diabetes.

**Methods:**

Consecutive patients undergoing elective CTO-PCI were prospectively enrolled from Apr. 2018 to May 2021. Patients were subdivided into 2 groups: Diabetes and No Diabetes. Detailed baseline characteristics, assessment of symptoms and QOL, angiographic and procedural details, in-hospital complications, and 1 month and 1 year follow-up data were collected. These data were analyzed accordingly for risk predictors of clinical outcome in patients who have diabetes and received successful CTO-PCI.

**Results:**

A total of 1076 patients underwent CTO-PCI attempts. Diabetes was present in 374 (34.76%) patients, who had more hypertension, previous PCI and stroke. Regarding the coronary lesions, diabetic patients suffered more LCX lesion, multivessel disease, number of lesions per patient, blunt stump, calcification and higher J-CTO score (p < 0.05). In-hospital major adverse cardiac event (MACE) (4.13% vs. 5.35%; p = 0.362) was similar in the two groups. At 1 month and 1 year follow-up after successful CTO-PCI, the incidence of MACE and all-cause mortality were also similar in the two groups (p > 0.05). Number of lesions per patient was an independent risk factor of MACE and all-cause mortality (p < 0.001) 1 year after successful CTO-PCI. Symptom and QOL were markedly improved regardless of diabetes both at 1 month and 1 year follow-up, and importantly, patients with diabetes showed similar degrees of improvement to those without diabetes (P > 0.05).

**Conclusions:**

Successful CTO-PCI could represent an effective strategy improving clinical outcome, symptoms and QOL in CTO patients with diabetes.

**Supplementary Information:**

The online version contains supplementary material available at 10.1186/s12933-022-01708-0.

## Introduction

Chronic total occlusion (CTO) accounted for approximately 13–41% of coronary artery disease (CAD) patients undergoing coronary angiography (CAG) [1, 2]. It has been reported that successful percutaneous coronary intervention (PCI) of CTO (CTO-PCI) could prolong long-term survival, relieve angina and dyspnea, and improve the ventricular function compared to failed revascularization [3–6]. Diabetes was a well-known CAD risk factor, and associated with a greater atherosclerotic burden, including diffused CAD, multivessel disease, and heavy coronary artery calcifications [7, 8]. Among the CTO patients, diabetes is relatively common, taking up approximately 30–40% [9, 10]. For CTO patients with diabetes, data regarding the clinical outcome after successful revascularization were controversial. Some reported that the incidence of long-term major adverse cardiac event (MACE) was higher in patients with diabetes [11, 12] while some found no obvious difference of long-term MACE in patients with or without diabetes [13–15]. Furthermore, as an important indicator for medical decision-making and a predictor for treatment success, quality of life (QOL) is therefore of prognostic importance. Especially for diabetic patients, QOL has been recognized as the ultimate goal [16, 17], while hitherto, there is no data regarding whether successful CTO-PCI improves QOL of CTO patients with diabetes. Therefore, in the present study, we aimed to comprehensively investigate the effect of successful CTO-PCI on clinical outcomes, symptoms and QOL, and to identify the variables associated with the incidence of the MACE and all-cause mortality in CTO patients with diabetes.

## Methods

### Patient population

**Fig. 1:**
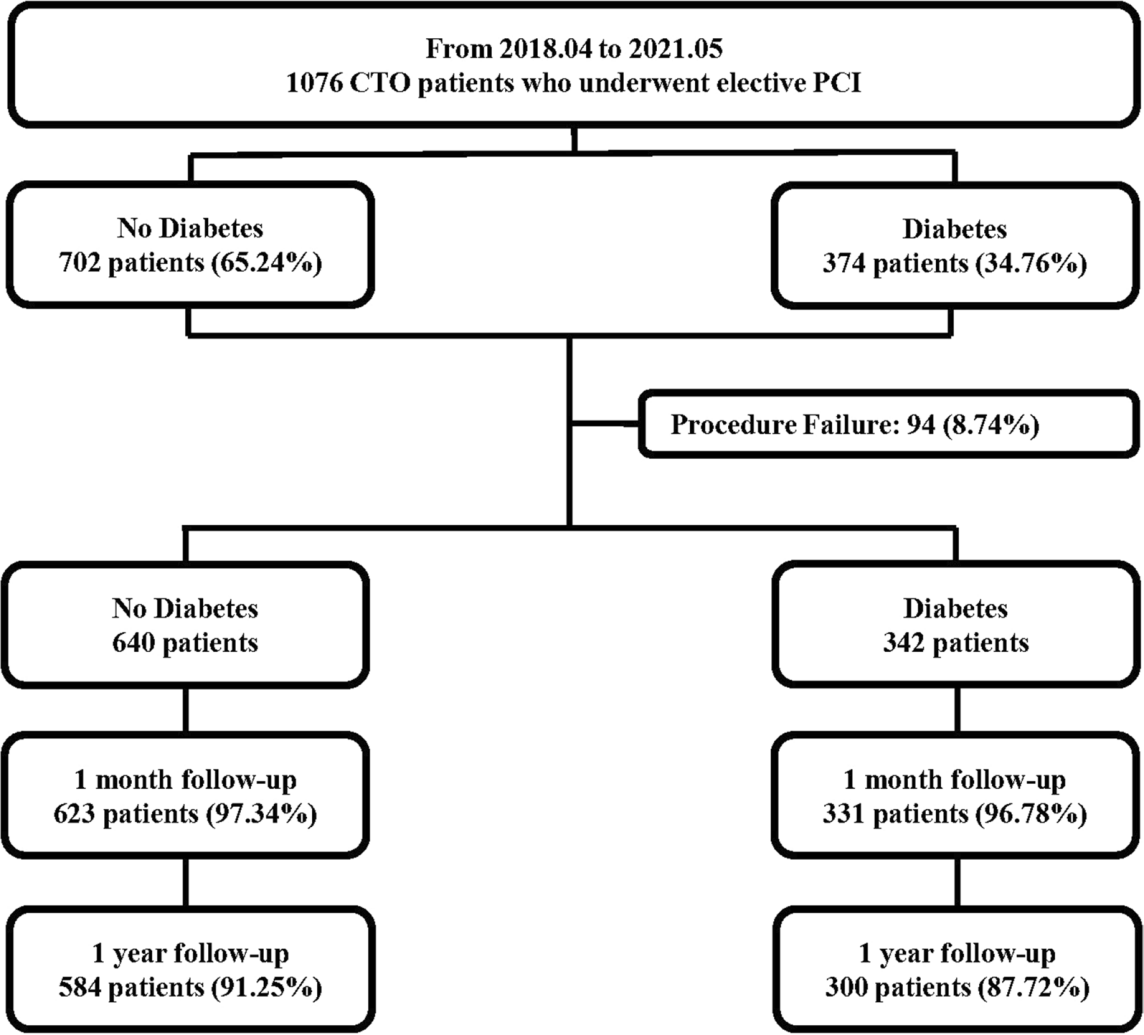
Flowchart of the Study Population. *CTO* chronic total occlusion, *PCI* percutaneous coronary intervention

A total of 1076 patients who underwent elective PCI for at least 1 CTO lesion from Apr. 2018 to May 2021 at Xijing Hospital were prospectively and consecutively enrolled in this analysis (Fig. 1). All the procedures were performed by one CTO team of Xijing Hospital which was led by Dr. Chengxiang Li. Importantly, all the procedures were completed by Dr. Li or under his guidance. Patients with acute myocardial infarctions (ST elevation or non-ST elevation), cardiogenic shock, and unstable hemodynamics were excluded. Indications for coronary revascularization were based on angina symptoms or on noninvasive imaging (coronary artery CT). The decision to the revascularization strategy (PCI or CABG, and lesions to be revascularized) for each patient rested with the cardiac surgeon and internationalists in our center. In case of surgical indication rejected by the patients, PCI was proposed if considered to be feasible by the international operator. Study population was divided into two groups based on whether or not the diabetes was present: diabetes (374, 34.76%) and no diabetes (702, 65.24%). The study was approved by the Ethics Committee of Xijing Hospital, the Fourth Military Medical University, and each subject was provided with informed consent before recruitment (KY20172019-1).

### Definition and endpoints

Coronary CTOs were defined as angiographic evidence of total occlusions with thrombolysis in myocardial infarction flow grade 0 within a major epicardial coronary artery of at least 2.5 mm, and estimated duration of at least 3 months. Non-CTO was defined as diameter stenosis 50% for left main (LM) and 70% for non-LM CAD within a vessel diameter ≥ 2.5 mm [18]. Diabetes was defined as a fasting plasma glucose level ≥ 7.0 mmol/L, or a plasma glucose level ≥ 11.1 mmol/L at 2 h after an oral glucose tolerance test, or glycated hemoglobin ≥ 6.5% on more than two occasions, or random plasma glucose value ≥ 11.1 mmol/L in presence of classic symptoms of hyperglycemia, or the current use of hypoglycemic agents or insulin [19]. Revascularization was considered for angiographically significant stenosis (≥ 70% diameter reduction by visual assessment) and functionally significant stenosis (fractional flow reserve measurement < 0.80). The complete revascularization was defined as revascularization in all major significantly diseased epicardial vessels during the same hospitalization. The J-CTO (Multicenter CTO Registry in Japan) [20]. Procedural success was defined as successful CTO revascularization with achievement of < 30% residual diameter stenosis within the treated segment and restoration of TIMI flow grade 3 antegrade, and without any in-hospital MACE. In-hospital MACE included any of the following adverse events prior to hospital discharge: all-cause mortality, nonfatal myocardial infarction (MI), and clinically driven revascularization. Major bleeding was defined as Bleeding Academic Research Consortium (BARC) type bleeding of least 3 [21].

### Follow-up

Patients were followed up by clinical visits or telephone interviews at 1 month and 1 year after CTO-PCI. The primary outcomes of interest for this study were the occurrence of MACE. MACE was defined as the composite of all-cause mortality, nonfatal MI and clinically driven revascularization by either PCI or CABG. The diagnosis of nonfatal MI was based on ECG findings (new Q waves in ≥ 2 contiguous leads) and cardiac biomarker elevations (more than 3 times the upper limit of creatine kinase or creatine kinase myocardial band in 2 plasma samples) [22]. Secondary endpoints were the change of symptoms and QOL across the three groups. In addition, details regarding MACE occurrence and health status assessment were obtained from hospital re-admission records, telephone contact with the referring physician, or outpatient visits.

### Symptoms assessment

Dyspnea was assessed at baseline, 1 month, and 1 year after CTO-PCI according to New York Heart Association (NYHA) functional class and Rose Dyspnea Scale (RDS), respectively. The RDS is a 4-item questionnaire to assesses patients’ level of dyspnea with common activities [23]. Each activity associated with dyspnea is assigned 1 point, where RDS scores range from 0 to 4, with a score of 0 indicating no dyspnea and increased scores indicating greater dyspnea.

The angina status of the patient was assessed according to the Seattle Angina Questionnaire (SAQ) [24] at baseline, 1 month, and 1 year after CTO-PCI. SAQ consists of 19 items that measure 5 dimensions: angina frequency (AF); angina stability (AS); disease perception (DP); physical limitation (PL) and treatment satisfaction (TS). All items use 5-point descriptive scales and scores are calculated by totalling all the single scores within each group and transforming them to a scale of 0 to 100, where 0 is the worst and 100 is the best.

### QOL assessment

Quality of life was assessed by means of the European Quality of Life-5 Dimensions (EQ-5D) and SF-12 questionnaire at baseline, 1 month, and 1 year after CTO-PCI. The EQ-5D assesses 5 dimensions of general health (mobility, self-care, usual activities, pain/discomfort, and anxiety/depression) with a 3-level scale. These scores can then be converted to utilities with an algorithm developed for Japan population. Utilities are preference-weighted health status assessments with scores that range from − 0.11 to 1.00, with 1.00 representing the perfectest health and − 0.11 representing the poorest health [25, 26].

The SF-12 is a shortened version of the SF-36, including 12 questions, with three to five answer categories each (Likert scale). The instrument covers eight dimensions: general health, physical functioning, role physical, bodily pain, vitality, social functioning, role emotional and mental health. Both a physical functioning component score (PCS) and a mental functioning component score (MCS), ranging between 0 and 100, can be calculated by using a scoring algorithm. Lower scores represent worse and higher scores represent better self-perceived HRQOL outcomes [27].

### Statistical analysis

The continuous variables are presented as mean ± SD or as medians and interquartile ranges. The categorical variables are presented as percentages. The continuous variables were compared using the t test or Mann–Whitney U test where appropriate, and categorical variables were compared using the chi-square or fisher’s exact test. The all-cause mortality was analyzed using the Kaplan Meier method. Logistic regression analysis was used for univariate and multivariate analysis. A 2-sided p-value of < 0.05 was considered significant. The IBM SPSS Statistics 25 and STATA MP 14.0 software were used for calculations.

## Results

A total of 1076 patients underwent CTO-PCI were prospectively and consecutively enrolled in the study, who were subdivided into 2 groups: diabetes (374, 34.76%), and no diabetes (702, 65.24%, Fig. 1). Compared to patients without diabetes, those with diabetes had more hypertension (58.83% vs. 67.11%; p = 0.008), previous PCI (48.15% vs. 55.61%; p = 0.020) and stroke (10.40% vs. 15.24%; p = 0.020), lower diastolic blood pressure, hemoglobin, CrCL, CK, and CK-MB level and higher FPG, NT-proBNP level (p < 0.05, Table 1). Additionally, the proportion of LCX lesion (68.80% vs. 81.02%; p < 0.001), multivessel disease (83.62% vs. 91.98%; p < 0.001), number of lesions per patient (2.40 ± 0.98 vs. 2.61 ± 0.93; p = 0.001), blunt stump (66.00% vs. 72.19%; p = 0.037), calcification (30.20% vs. 38.50%; p = 0.006) and J-CTO score (2.11 ± 1.13 vs. 2.31 ± 1.15; p = 0.005) were significantly higher in patients with diabetes than those without diabetes (Table 2). The incidence of in-hospital MACE was similar in patients with diabetes or not (p > 0.05, Table 3).

**Table 1:** Baseline Characteristics

	No Diabetes	Diabetes	p Value
	(n = 702)	(n = 374)	
Age, yrs	60.23 ± 10.78	61.35 ± 10.55	0.101
Males, n%	609(86.75)	333(89.04)	0.280
BMI, kg/m^2^	25.25 ± 3.25	25.11 ± 3.03	0.474
SBP, mmHg	127.70 ± 20.23	126.96 ± 19.25	0.560
DBP, mmHg	72.87 ± 12.19	71.19 ± 11.99	0.031
Smoking, n%	275(39.17)	125(33.42)	0.063
Hypertension, n%	413(58.83)	251(67.11)	0.008
Previous MI, n%	298(42.45)	145(38.77)	0.243
Previous PCI, %	338(48.15)	208(55.61)	0.020
Previous CABG, %	20(2.85)	15(4.01)	0.306
Previous stroke, n%	73(10.40)	57(15.24)	0.020
Chronic pulmonary disease, n%	13(1.85)	9(2.41)	0.540
Peripheral artery disease, n%	14(1.99)	13(3.48)	0.139
Family history of CHD, n%	23(3.28)	21(5.61)	0.065
WBC, *10^9^/L	6.98 ± 2.09	6.94 ± 1.97	0.783
Platelet, *10^9^/L	208.63 ± 64.78	206.42 ± 65.43	0.596
Hemoglobin, g/L	141.24 ± 19.35	136.64 ± 18.96	< 0.001
FPG, mmol/ L	5.55 ± 1.82	8.09 ± 3.53	< 0.001
TC, mmol/ L	3.44 ± 1.06	3.28 ± 0.91	0.008
TG, mmol/ L	1.67 ± 1.02	1.66 ± 1.23	0.917
LDL-C, mmol/ L	1.96 ± 0.93	1.78 ± 0.77	0.001
HDL-C, mmol/ L	1.03 ± 0.42	0.98 ± 0.34	0.087
ALT, U/L	33.56 ± 47.35	28.73 ± 25.79	0.068
AST, U/L	29.35 ± 43.62	23.80 ± 19.81	0.004
Scr, μmol/ L	89.24 ± 64.23	94.90 ± 55.80	0.151
eGFR, mL/min per 1.73 m^2^	83.34 ± 23.22	80.40 ± 27.85	0.081
CrCL, ml/min	88.17 ± 30.37	84.03 ± 32.68	0.040
Uric acid, μmol/ L	355.90 ± 95.27	337.98 ± 105.21	0.005
Hyperuricemia, n%	145(20.66)	63(16.84)	0.132
cTnI, ng/mL	1.01 ± 9.32	0.62 ± 3.93	0.433
NT-proBNP, pg/ml	294.80(115.00,956.93)	368.60(127.90,1264.00)	0.040
CK, IU/L	146.16 ± 343.72	106.19 ± 123.31	0.010
CK-MB, IU/L	18.58 ± 34.10	14.76 ± 14.60	0.019
LVEF, %	50.53 ± 9.47	49.46 ± 10.25	0.096
Dyspnea(NYHA functional class), n%			0.273
I	194(27.64)	96(25.67)	
II	340(45.58)	180(48.13)	
III	145(20.66)	75(20.05)	
IV	23(3.28)	23(6.15)	
NYHA functional class III/ IV	168(23.93)	98(26.20)	0.411

*BMI* body mass index, *SBP* systolic blood pressure, *DBP* diastolic blood pressure, *MI* myocardial infarction, *PCI* percutaneous coronary intervention, *CABG* coronary artery bypass grafting, *CHD* coronary atherosclerotic heart disease, *WBC* white blood cell, *FPG* fasting plasma glucose, *TC* total cholesterol, *TG* triglyceride, *LDL-C* low density lipoprotein cholesterol, *HDL-C* high density lipoprotein cholesterol, *ALT* alanine aminotransaminase, *AST* aspartate aminotransferase, *Scr* serum creatinine, *eGFR* estimated glomerular filtration rate, *CrCL* creatinine clearance, *cTnI* cardiac troponin I, *NT-proBNP* N-terminal pro-B type natriuretic peptide, *CK* creatine kinase, *CK-MB* creatine kinase MB, *LVEF* left ventricular ejection fraction, *NYHA* New York Heart Association

**Table 2:** Angiographic Characteristics and Procedural Details

	No Diabetes	Diabetes	p Value
	(n = 702)	(n = 374)	
Vascular lesion, n%			
LM lesion	129(18.38)	82(21.93)	0.163
LAD lesion	578(82.34)	323(86.36)	0.088
LCX lesion	483(68.80)	303(81.02)	< 0.001
RCA lesion	552(78.63)	301(80.48)	0.476
Multivessel disease, n%	587(83.62)	344(91.98)	< 0.001
Number of lesions per patient	2.40 ± 0.98	2.61 ± 0.93	0.001
Location of the CTO, n%			
LM-CTO	7(1.00)	3(0.80)	0.751
LAD-CTO	327(46.58)	169(45.19)	0.662
LCX-CTO	202(28.77)	120(32.09)	0.259
RCA-CTO	403(57.41)	232(62.03)	0.142
Multi-CTO lesion, n%	205(29.20)	126(33.69)	0.129
Number of CTO per patient	1.34 ± 0.56	1.40 ± 0.61	0.087
CTO target vessel, n%			
LM-CTO	5(0.71)	3(0.80)	0.870
LAD-CTO	272(38.75)	141(37.70)	0.737
LCX-CTO	107(15.24)	52(13.90)	0.556
RCA-CTO	347(49.43)	192(51.34)	0.551
Ostial location, n%	68(9.69)	39(10.43)	0.699
In-stent occlusion, n%	50(7.12)	39(10.43)	0.061
Lesion length, mm	27.85 ± 20.57	28.39 ± 16.96	0.672
Lesion length ≥ 20 mm, n%	450(64.10)	255(68.18)	0.180
Blunt stump, n%	463(66.00)	270(72.19)	0.037
Tortuosity ≥ 45°, n%	194(27.64)	112(29.95)	0.424
Calcification, n%	212(30.20)	144(38.50)	0.006
Reattempt, n%	104(14.81)	66(17.65)	0.225
J-CTO score	2.11 ± 1.13	2.31 ± 1.15	0.005
Proximal cap side-branch, n%	526(74.93)	287(76.74)	0.511
“Interventional” collaterals, n%	496(70.66)	266(71.12)	0.809
Diseased distal landing zone, n%	335(47.72)	200(53.48)	0.072
Contrast volume, ml	363.64 ± 203.53	362.99 ± 245.91	0.963
Procedural time, min	119.14 ± 69.29	117.21 ± 69.97	0.667
Procedural success, n%	640(91.17)	342(91.44)	0.879

*LM* left main coronary artery, LAD left anterior descending coronary artery, *LCX* left circumtrunnion coronary artery, *RCA* right coronary artery, *CTO* chronic total occlusion, *J-CTO* multicenter CTO registry in Japan

**Table 3:** In-hospital MACE

	No Diabetes	Diabetes	p Value
	(n = 702)	(n = 374)	
MACE, n%	29(4.13)	20(5.35)	0.362
All-cause mortality, n%	4(0.57)	3(0.80)	0.652
Cardiac mortality, n%	4(0.57)	3(0.80)	0.652
Nonfatal MI, n%	12(1.71)	5(1.34)	0.641
Clinically driven revascularization, n%	14(1.99)	13(3.48)	0.139
Emergency PCI	14(1.99)	13(3.48)	0.139
Emergency CABG	0	0	−

*MACE* major adverse cardiac event, *MI* myocardial infarction, *PCI* percutaneous coronary intervention, *CABG* coronary artery bypass grafting

**Fig. 2:**
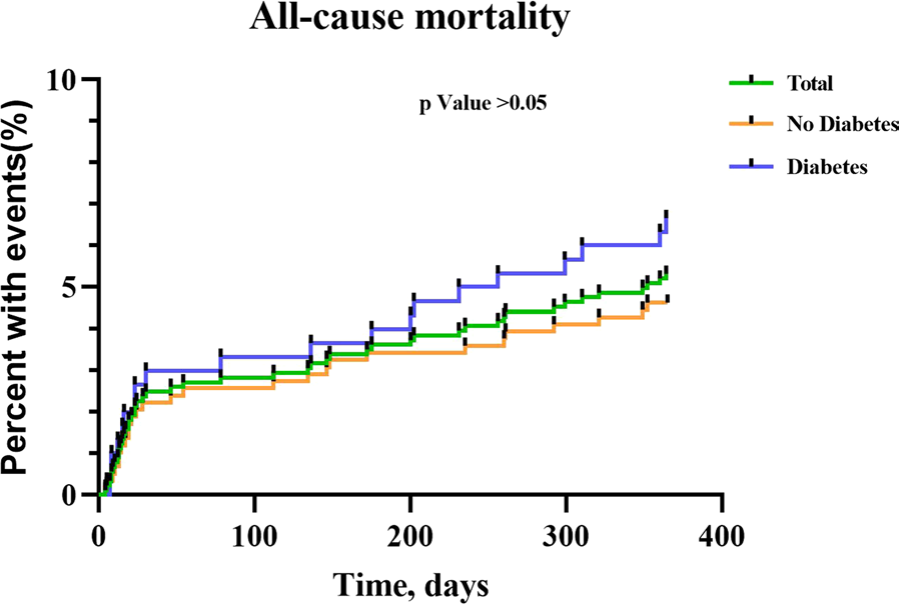
Kaplan–Meier analysis of all-cause mortality in Patients with Successful CTO-PCI. *CTO* chronic total occlusion, *PCI* percutaneous coronary intervention

**Fig. 3:**
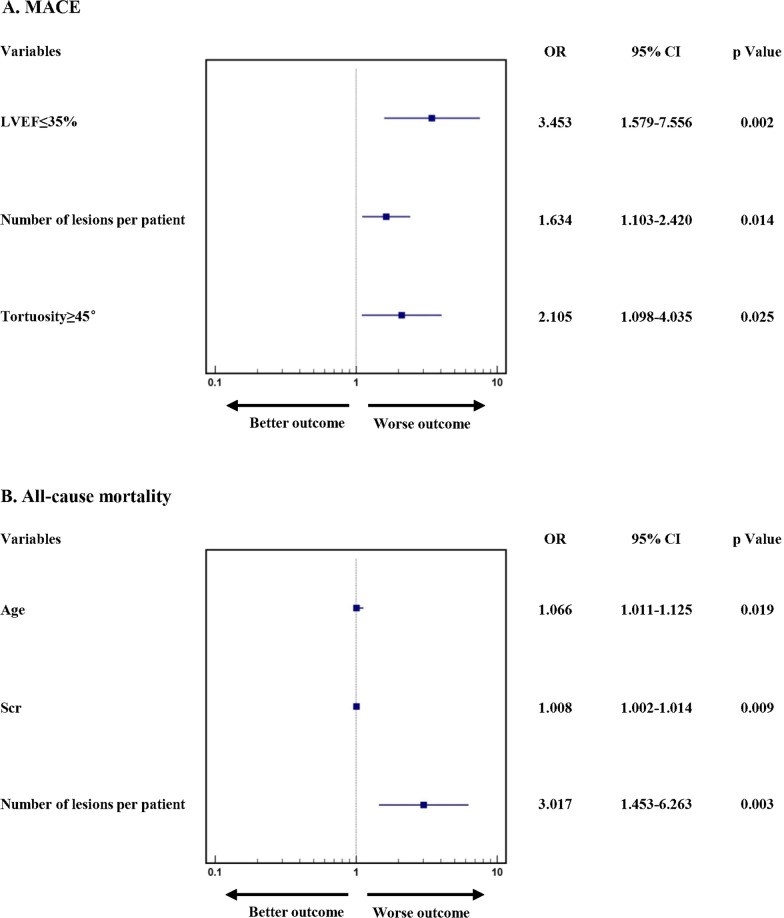
Multivariable Logistic Regression for 1-year Clinical Outcomes in Diabetic Patients with Successful CTO-PCI. *CTO* chronic total occlusion, *PCI* percutaneous coronary intervention, *LVEF* left ventricular ejection fraction, *Scr* serum creatinine, *OR* odds ratio, *CI* confidential interval

After successful CTO-PCI, follow-up visits were carried out at 1 month, completed for 623 (97.34%) and 331 (96.78%), and 1 year, completed for 584 (91.25%) and 300 (87.72%). The occurrence of MACE, including its three sub-items, was similar in the two groups both at 1 month (8.67% vs. 11.48%; p = 0.161) and 1 year (13.36% vs. 17.76%; p = 0.088, Table 4, Fig. 2). Moreover, the univariable and multivariable analysis of the CTO patients with diabetes receiving successful CTO-PCI showed that LVEF ≤ 35% (OR: 3.453, 95% CI 1.579–7.556, p = 0.002), number of lesions per patient (OR: 1.634, 95% CI 1.103–2.420, p = 0.014) and tortuosity ≥ 45° (OR: 2.105, 95% CI 1.098–4.035, p = 0.025) independently increased the risk of 1 year MACE (Table S4A, Fig. 3A). And, Age (OR: 1.066, 95% CI 1.011–1.125, p = 0.019), higher Scr (OR: 1.008, 95% CI 1.002–1.014, p = 0.009) and number of lesions per patient (OR: 3.017, 95% CI 1.453–6.263, p = 0.003) were independent risk factors of 1-year all-cause mortality (Table S4B, Fig. 3B).

**Table 4:** Clinical Outcomes of Patients with Successful CTO-PCI

	No Diabetes	Diabetes	p Value
1 month follow-up	623	331	
MACE, n%	54(8.67)	38(11.48)	0.161
All-cause mortality, n%	13(2.09)	9(2.72)	0.536
Nonfatal MI, n%	6(0.96)	3(0.91)	0.931
Clinically driven revascularization, n%	39(6.26)	29(8.76)	0.153
1 year follow-up	584	300	
MACE, n%	78(13.36)	53(17.76)	0.088
All-cause mortality, n%	27(4.62)	20(6.67)	0.200
Nonfatal MI, n%	13(2.23)	6(2.00)	0.826
Clinically driven revascularization, n%	50(8.56)	36(12.00)	0.102

*CTO* chronic total occlusion, *PCI* percutaneous coronary intervention, *MACE* major cardiac event, *MI* myocardial infarction

**Fig. 4:**
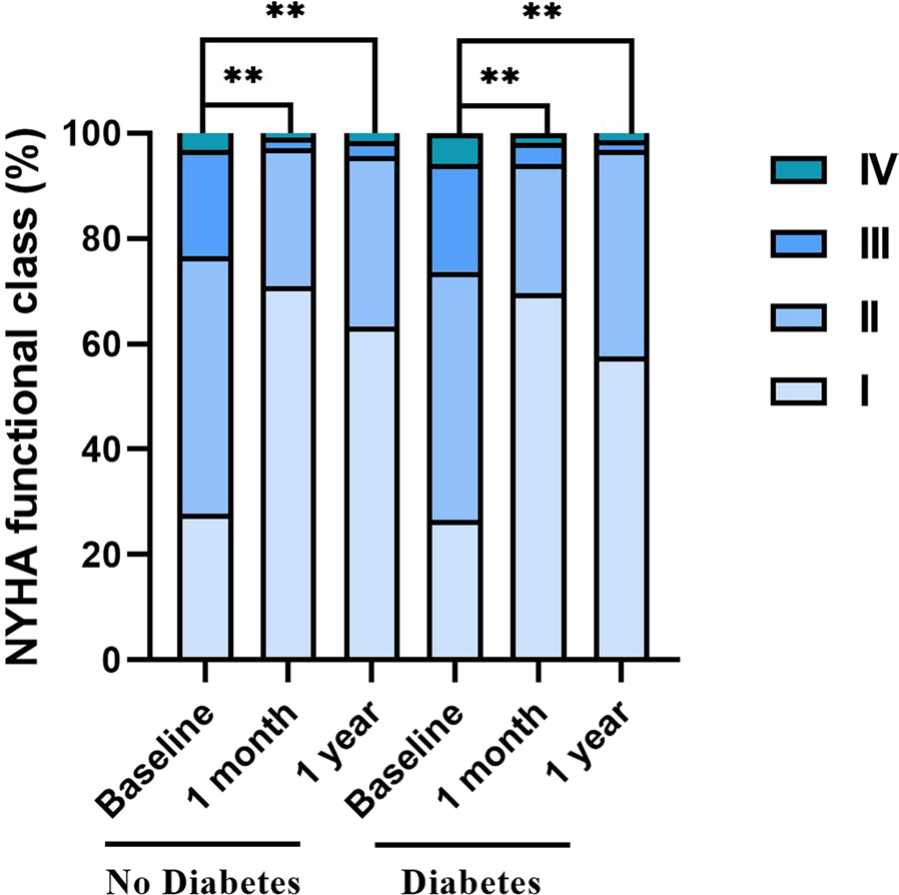
Changes of NYHA Functional Class in Patients with Successful CTO-PCI. ^**^ p Value < 0.001. *NYHA* New York Heart Association*, CTO* chronic total occlusion, *PCI* percutaneous coronary intervention

In regard to symptoms, significant improvement of dyspnea and angina were observed in all the CTO patients with successful CTO-PCI at 1 month and 1 year follow-up (Fig. 4 and Table 5). Compared with baseline, the proportion of NYHA functional class III/ IV and their RDS scores of the two groups was obviously decreased at 1 month and 1 year after CTO-PCI (p < 0.001, Fig. 4, Table 5); notably, RDS score in patients with diabetes decreased at a similar degree to those without diabetes (p > 0.05, Table 5), suggesting that successful CTO-PCI significantly alleviated dyspnea of all patients. Additionally, for patients with angina, successful CTO-PCI markedly increased the SAQ-AS and SAQ-AF scores of the two groups both at 1 month and 1 year follow-up (p < 0.001), and the SAQ-AS and SAQ-AF scores increased at the similar degree in the two groups (p > 0.05, Table 6), indicating that successful CTO-PCI also greatly relieved the angina of patients with diabetes or not.

**Table 5:** Changes of Symptoms in Patients with Successful CTO-PCI Comparison of changes in RDS from baseline to follow-up

	RDS
No Diabetes	
Baseline	1.03 ± 1.10
1 month follow-up	0.21 ± 0.47
1 year follow-up	0.46 ± 0.65
^m^Δ	0.81 ± 1.17
^y^Δ	0.57 ± 1.21
^m^p Value	< 0.001
^y^p Value	< 0.001
Diabetes	
Baseline	1.06 ± 1.08
1 month follow-up	0.25 ± 0.63
1 year follow-up	0.53 ± 0.71
^m^Δ	0.80 ± 1.22
^y^Δ	0.52 ± 1.29
^m^p Value	< 0.001
^y^p Value	< 0.001
^a^p Value	0.855
^b^p Value	0.654

*CTO* chronic total occlusion, *PCI* percutaneous coronary intervention, *RDS* Rose Dyspnea Scale

^m^Δ change between baseline and 1 month follow-up

^y^Δ change between baseline and 1 year follow-up

^m^p Value: baseline versus 1 month follow-up

^y^p Value: baseline versus 1 year follow-up

^a^p Value: comparison of ^m^Δ in the two groups

^b^p Value: comparison of ^y^Δ in the two groups

**Table 6:** Comparison of changes in SAQ subscales from baseline to follow-up

	SAQ-PL	SAQ-AS	SAQ-AF	SAQ-TS	SAQ-DP
No Diabetes					
Baseline	63.49 ± 14.59	50.23 ± 17.19	78.86 ± 24.38	79.93 ± 14.78	69.58 ± 18.74
1 month follow-up	69.12 ± 12.98	70.78 ± 23.97	97.93 ± 7.86	83.47 ± 11.56	72.31 ± 13.83
1 year follow-up	67.78 ± 11.95	71.95 ± 27.15	94.15 ± 11.21	86.65 ± 10.54	78.73 ± 15.62
^m^Δ	5.46 ± 19.02	20.74 ± 30.16	19.56 ± 24.99	3.34 ± 16.45	2.90 ± 21.52
^y^Δ	4.69 ± 17.89	20.00 ± 32.68	16.48 ± 25.91	6.26 ± 18.37	9.47 ± 25.16
^m^p Value	< 0.001	< 0.001	< 0.001	< 0.001	0.005
^y^p Value	< 0.001	< 0.001	< 0.001	< 0.001	< 0.001
Diabetes					
Baseline	61.72 ± 16.10	50.00 ± 15.08	76.67 ± 26.07	80.25 ± 15.47	70.54 ± 19.01
1 month follow-up	67.33 ± 14.45	72.90 ± 23.87	98.26 ± 7.07	84.16 ± 11.09	71.22 ± 14.18
1 year follow-up	66.68 ± 14.04	70.09 ± 29.03	94.61 ± 11.88	87.08 ± 9.31	78.51 ± 16.04
^m^Δ	5.31 ± 20.37	22.98 ± 27.69	22.02 ± 26.32	3.76 ± 17.82	0.98 ± 22.74
^y^Δ	5.46 ± 18.78	20.00 ± 32.68	20.04 ± 29.83	6.53 ± 19.31	8.30 ± 26.45
^m^p Value	< 0.001	< 0.001	< 0.001	< 0.001	0.617
^y^p Value	< 0.001	< 0.001	< 0.001	< 0.001	< 0.001
^a^p Value	0.911	0.267	0.161	0.715	0.206
^b^p Value	0.562	0.389	0.076	0.843	0.534

*SAQ* seattle angina questionnaire, *SAQ-PL* seattle angina questionnaire-physical limitation, *SAQ-AS* seattle angina questionnaire-anginal stability, *SAQ-AF* seattle angina questionnaire-anginal frequency, *SAQ-TS* seattle angina questionnaire-treatment satisfaction, SAQ-DP seattle angina questionnaire-disease perception

^m^Δ change between baseline and 1 month follow-up

^y^Δ change between baseline and 1 year follow-up

^m^p Value: baseline versus 1 month follow-up

^y^p Value: baseline versus 1 year follow-up

^a^p Value: comparison of ^m^Δ in the two groups

^b^p Value: comparison of ^y^Δ in the two groups

Most importantly, EQ-5D and SF-12 were firstly used to assess QOL of CTO patients with diabetes in the present study. Compared with baseline, PCS score of the two groups was greatly elevated at 1 month and 1 year follow-up (p < 0.001, Table 7) and the two groups showed similar improvement of PCS score (p > 0.05, Table 7). Likewise, EQ-5D score also exhibited a significant increase at 1 month and 1 year after successful CTO-PCI both in the patients with diabetes or without (p < 0.001, Table 8). These data suggested successful CTO-PCI remarkably improved QOL and the degree of improvement in patients with diabetes was similar to those without diabetes.

**Table 7:** Changes of Quality of Life in Patients with Successful CTO-PCI SF-12 during follow-up in each group

	**PCS**	**MCS**
No Diabetes		
Baseline	44.73 ± 8.11	53.96 ± 6.95
1 month follow-up	50.42 ± 6.13	56.24 ± 4.69
1 year follow-up	51.07 ± 6.54	54.75 ± 6.80
^m^Δ	5.53 ± 9.99	2.28 ± 7.93
^y^Δ	6.34 ± 10.22	0.64 ± 9.47
^m^p Value	< 0.001	< 0.001
^y^p Value	< 0.001	0.081
Diabetes		
Baseline	44.00 ± 8.44	54.50 ± 7.29
1 month follow-up	50.16 ± 7.06	55.51 ± 1.52
1 year follow-up	51.07 ± 6.54	54.16 ± 6.78
^m^Δ	5.92 ± 10.15	1.13 ± 8.19
^y^Δ	7.19 ± 10.28	-0.29 ± 10.54
^m^p Value	< 0.001	0.015
^y^p Value	< 0.001	0.722
^a^p Value	0.576	0.038
^b^p Value	0.257	0.197

*CTO* chronic total occlusion, *PCI* percutaneous coronary intervention, *PCS* physical functioning component score, *MCS* mental functioning component score

^m^Δ change between baseline and 1 month follow-up

^y^Δ change between baseline and 1 year follow-up

^m^p Value: baseline versus 1 month follow-up

^y^p Value: baseline versus 1 year follow-up

^a^p Value: comparison of ^m^Δ in the two groups

^b^p Value: comparison of ^y^Δ in the two groups

**Table 8:** EQ-5D during follow-up in each group

	**EQ-5D**
No Diabetes	
Baseline	0.89 ± 0.16
1 month follow-up	0.97 ± 0.09
1 year follow-up	0.97 ± 0.09
^m^Δ	0.08 ± 0.18
^y^Δ	0.07 ± 0.18
^m^p Value	< 0.001
^y^p Value	< 0.001
Diabetes	
Baseline	0.88 ± 0.18
1 month follow-up	0.95 ± 0.12
1 year follow-up	0.95 ± 0.13
^m^Δ	0.07 ± 0.20
^y^Δ	0.06 ± 0.22
^m^p Value	< 0.001
^y^p Value	< 0.001
^a^p Value	0.602
^b^p Value	0.378

*CTO* chronic total occlusion, *PCI* percutaneous coronary intervention, *EQ-5D* European Quality of Life-5 Dimensions

^m^Δ change between baseline and 1 month follow-up

^y^Δ change between baseline and 1 year follow-up

^m^p Value: baseline versus 1 month follow-up

^y^p Value: baseline versus 1 year follow-up

^a^p Value: comparison of ^m^Δ in the two groups

^b^p Value: comparison of ^y^Δ in the two groups

## Discussion

This is the first prospective study to comprehensively evaluate the effect of successful revascularization on clinical outcomes, symptoms and QOL for CTO patients with diabetes. The main findings of the present study were as follows: (1) The CTO patients combined with diabetes had more hypertension, previous PCI and stroke, multivessel disease, number of lesions per patient and higher J-CTO score; (2) the occurrence of in-hospital MACE was similar in CTO patients with or without diabetes; (3) the incidence of MACE and all-cause mortality were similar in CTO patients with or without diabetes after successful CTO-PCI both at 1 month and 1 year follow-up; (4) LVEF ≤ 35%, number of lesions per patient and tortuosity ≥ 45° independently increased the risk of 1 year MACE and age, higher Scr level and number of lesions per patient were independent risk factors of 1-year all-cause mortality for diabetic patients underwent successful CTO-PCI; (5) successful CTO-PCI significantly alleviated symptoms and improved QOL regardless of diabetes, at similar rate across the two groups. This study demonstrated the benefits of successful CTO-PCI in patients with diabetes in a real-world setting (See Additional file 1: Table S1–S4).

China has the highest number of diabetic patients and annual number of deaths from diabetes, at approximately 140.9 and 1.4 million respectively [28]. In our study, diabetes was found with an overall prevalence of 34.76% in CTO patients, consistent with the epidemiology in the North America [9, 10]. Previous studies reported that diabetes was associated with greater burden of comorbidity, longer lesions, and more complex anatomy in CTO patients [7, 8, 29]. In the present study, we found CTO patients with diabetes suffered more hypertension, previous PCI and stroke, multivessel disease, number of lesions per patient, blunt stump, calcification and higher J-CTO score.

The benefits of clinical outcome of CTO patients combined with diabetes after successful CTO-PCI was still controversial. Guo L et al. found that the incidence of MACE was significantly higher in patients with diabetes after successful CTO-PCI at a 2.6-year follow-up [11]. Sanguineti F et al. also reported that CTO patients with diabetes suffered more MACE at the median follow-up 4.2 years [12]. However, there also existed some studies found that the MACE in patients with or without diabetes was not of obvious difference at the 1.7–5-year follow-up [13–15], importantly, all of which were from the Asia and consistent with our findings. In our study, the occurrence of MACE, including all-cause mortality, nonfatal MI and clinically driven revascularization, was similar in the CTO patients no matter whether combined with diabetes or not both at 1 month and 1 year after successful CTO-PCI. These findings demonstrate the necessity to achieve revascularization in these diabetic patients to achieve better clinical outcome.

For CHD patients, especially those suffered from CTO lesion, severe adverse symptoms, such as angina and dyspnea, have been the most troubling problems, hence symptoms improvement has been proposed as the primary indication for CTO-PCI by Global Expert Consensus [30]. Many RCT, registry and observational studies have demonstrated that successful CTO-CPI could improve symptoms, including angina and dyspnea [31–34]. However, to date, only one study by Salisbury AC et al. reported that successful CTO-PCI relieved angina and dyspnea and of a similar magnitude regardless of diabetes status at 1 year follow-up [29]. Similarly, our study also found that successful CTO-PCI could greatly alleviate symptoms of patients with diabetes at a comparable degree to those without diabetes.

QOL has been defined by WHO as people’ “perceptions of their position in life in the context of culture and value systems in which they live, and in relation to their goals, expectations, standards, and concerns”; it involves many important domains of human dynamics such as the physical, psychological, social, environmental, and spiritual factors [35]. Therefore, QOL is receiving increasing attention recently due to its critical status in assessing patients’ well-being. Additionally, diabetic patients require life-long self-care, including the improvement of both long-term health and QOL, indicating QOL is the ultimate goal [16, 17]. However, hitherto, no study has evaluated the impact of successful revascularization on QOL for CTO patients with diabetes. In the present study, we firstly assessed QOL of these patients using well-recognized QOL questionnaire SF-12 and EQ-5D, and found that successful CTO-PCI could greatly improve QOL of diabetic patients at a similar degree between patients with and without diabetes.

## Study limitations

This study undoubtedly has some limitations. First, the single-team nature of our study is a potential weakness, which may be not suitable for other centers or team. Second, no angiographic and echocardiography follow-up data were collected. Third, our study did not include those who were either not provided PCI or referred for surgical revascularization. Fourth, we have no accessible information on residual ischemia. Fifth, objective measurements of physical capacities, such as those from exercise stress testing were not systematically available in follow-up. Finally, noninvasive testing such as cardiac magnetic resonance imaging should be performed in patients to assess the myocardial viability, which will be carried out in the future work.

## Conclusions

The present study demonstrates that timely successful CTO-PCI was necessary for patients with diabetes to bring reduced long-term MACE, substantial symptom alleviation and improved QOL.

## Supplementary Information

**Additional file 1**: **Table S1 **Non-CTO lesion characteristics. **Table S2 **Intraprocedural and In-hospital Complications. **Table S3 **Univariable Logistic Regression for 1-month Clinical Outcomes in Diabetic Patients with Successful CTO-PCI. **Table S4 **Univariable Logistic Regression for 1-year Clinical Outcomes in Diabetic Patients with Successful CTO-PCI

## Data Availability

The datasets generated and analyzed for this current study are available from the corresponding author upon reasonable request.

## Abbreviations

ALT: Alanine aminotransaminase
AST: Aspartate aminotransferase
BMI: Body mass index
CABG: Coronary artery bypass graft
CHD: Coronary atherosclerotic heart disease
CI: Confidence interval
cTnI: Cardiac troponin I
CTO: Chronic total occlusion
CTO-PCI: Chronic total occlusion treated with percutaneous coronary intervention
DBP: Diastolic blood pressure
eGFR: estimated glomerular filtration rate
EQ-5D: European quality of life-5 dimensions
FPG: Fasting plasma glucose
HDL-C: High density lipoprotein cholesterol
HF: Heart failure
J-CTO: Multicenter CTO registry in Japan
LAD: Left anterior descending coronary artery
LCX: Left circumtrunnion coronary artery
LDL-C: Low density lipoprotein cholesterol
LM: Left main coronary artery
LVEF: Left ventricular ejection fraction
MACE: Major adverse cardiac event
MCS: Mental functioning component score
MI: Myocardial infarction
NT-proBNP: N-terminal pro-B type natriuretic peptide
NYHA: New York Heart Association
OR: Odds ratio
PCI: Percutaneous coronary intervention
PCS: Physical functioning component score
QOL: Quality of life
RCA: Right coronary artery
RCT: Randomized controlled trial
RDS: Rose dyspnea scale
SAQ: Seattle Angina Questionnaire
SAQ-PL: Seattle Angina Questionnaire-physical limitation
SAQ-AS: Seattle Angina Questionnaire-anginal stability
SAQ-DP: Seattle Angina Questionnaire-disease perception
SAQ-AF: Seattle Angina Questionnaire-anginal frequency
SAQ-TS: Seattle Angina Questionnaire-treatment satisfaction
SBP: Systolic blood pressure
Scr: Serum creatinine
TC: Total cholesterol
TG: Triglyceride
WBC: white blood cell
