# Supplementary material for: Benefits of successful percutaneous coronary intervention in chronic total occlusion patients with diabetes
Source: Cardiovasc Diabetol. 2022 Dec 5;21:271. doi: 10.1186/s12933-022-01708-0 (PMC9724402; doi:10.1186/s12933-022-01708-0)
Supplement: Supplementary file 1 — Additional file 1: Table S1 Non-CTO lesion characteristics. Table S2 Intraprocedural and In-hospital Complications. Table S3 Univariable Logistic Regression for 1-month Clinical Outcomes in Diabetic Patients with Successful CTO-PCI. Table S4 Univariable Logistic Regression for 1-year Clinical Outcomes in Diabetic Patients with Successful CTO-PCI [file 12933_2022_1708_MOESM1_ESM.docx]

**Additional file**

**Supplementary Result**

Non-CTO lesion characteristics and the treatment of the non-CTO lesion of patients are showed in Table S1. The proportion of LCX lesion was the higher in patients with diabetes than those without diabetes (p=0.011).

Regarding the intraprocedural and in-hospital complications, the occurrence of intraprocedural and in-hospital complications were similar in CTO patients with diabetes or not (p>0.05, Table S2).

Multivariable logistic analysis identified that previous MI (OR: 2.079, 95% CI: 1.035-4.176, p=0.040) and LVEF**≤**35% (OR: 3.403, 95% CI: 1.462-7.918, p=0.004) were independent risk factors of 1 month MACE (Table S3A). And lower eGFR (OR: 0.972, 95% CI: 0.946-0.999, p=0.042) was an independent risk factors of 1-month all-cause mortality (Table S3B).

**Additional file Table**

**Additional file 1: Table S1 Non-CTO lesion characteristics**

|  | **No Diabetes** | **Diabetes** | **p Value** |
| --- | --- | --- | --- |
|  | **(n= 702)** | **(n= 374)** |  |
| **Location of the non-CTO, n%** |  |  |  |
| **LM** | 122(17.38) | 79(21.12) | 0.133 |
| **LAD** | 251(35.75) | 154(41.18) | 0.080 |
| **LCX** | 281(40.03) | 180(48.13) | 0.011 |
| **RCA** | 149(21.23) | 69(18.45) | 0.281 |
| **Non-CTO target vessel, n%** |  |  |  |
| **LM** | 44(6.27) | 27(7.22) | 0.549 |
| **LAD** | 116(16.52) | 68(18.18) | 0.492 |
| **LCX** | 81(11.54) | 43(11.50) | 0.984 |
| **RCA** | 69(9.83) | 40(10.70) | 0.654 |
| **Complete revascularization, n%** | 231(32.91) | 85(22.73) | <0.001 |

*CTO* chronic total occlusion, *LM* left main coronary artery, *LAD* left anterior descending coronary artery *LCX* left circumtrunnion coronary artery, *RCA* right coronary artery

**Additional file 1: Table S2 Intraprocedural and In-hospital Complications**

|  | **No Diabetes** | **Diabetes** | **p Value** |
| --- | --- | --- | --- |
|  | **(n= 702)** | **(n= 374)** |  |
| **Intraprocedural complications** | 27(3.85) | 14(3.74) | 0.933 |
| **All-cause mortality, n%** | 2(0.28) | 0(0) | 0.302 |
| **Cardiac mortality, n%** | 2(0.28) | 0(0) | 0.302 |
| **Cardiac arrest, n%** | 1(0.14) | 0(0) | 0.465 |
| **Malignant arrhythmia, n%** | 0 | 0 | - |
| **Pericardial tamponade, n%** | 2(0.28) | 0(0) | 0.302 |
| **Pericardiocentesis, n%** | 1(0.14) | 0(0) | 0.465 |
| **stroke, n%** | 0 | 0 | - |
| **Vascular dissection, n%** | 14(1.99) | 9(2.41) | 0.656 |
| **Collateral vessel perforation, n%** | 4(0.57) | 2(0.53) | 0.941 |
| **Loss of side branch≥2mm, n%** | 8(1.14) | 2(0.53) | 0.325 |
| **Acute renal failure, n%** | 0 | 0 | - |
| **Contrast allergy-related shock, n%** | 0 | 0 | - |
| **Vasovagal response, n%** | 0 | 1(0.27) | 0.170 |
| **In-hospital complications** | 77(10.97) | 55(14.71) | 0.075 |
| **MACE** | 29(4.13) | 20(5.35) | 0.362 |
| **Other complications** | 60(8.55) | 45(12.03) | 0.067 |
| **Pericardiocentesis, n%** | 3(0.43) | 3(0.80) | 0.432 |
| **Cardiac arrest, n%** | 3(0.43) | 5(1.34) | 0.098 |
| **Malignant arrhythmia, n%** | 4(0.57) | 6(1.60) | 0.092 |
| **Cardiac shock, n%** | 8(1.14) | 2(0.53) | 0.325 |
| **Major bleeding, n%** | 38(5.41) | 24(6.42) | 0.501 |
| **Allergy-related shock, n%** | 2(0.28) | 0(0) | 0.302 |
| **Acute renal failure, n%** | 3(0.43) | 2(0.5) | 0.805 |
| **Subcutaneous hematoma, n%** | 23(3.28) | 14(3.74) | 0.689 |
| **Vasovagal response, n%** | 4(0.57) | 9(2.41) | 0.009 |

*MACE* major adverse cardiac event

**Additional file 1: Table S3 Univariable Logistic Regression for 1-month Clinical Outcomes in Diabetic Patients with Successful CTO-PCI**

**A. MACE**

|  | **Univariable analysis** | | | **Multivariable analysis** | | |
| --- | --- | --- | --- | --- | --- | --- |
|  | **OR** | **95%CI** | **p Value** | **OR** | **95%CI** | **p Value** |
| **Previous MI** | 2.313 | 1.168-4.580 | 0.016 | 2.079 | 1.035-4.176 | 0.040 |
| **LVEF≤35%** | 3.829 | 1.669-8.782 | 0.002 | 3.403 | 1.462-7.918 | 0.004 |
| **Multi-CTO lesion** | 1.998 | 1.009-3.956 | 0.047 |  |  |  |

*CTO* chronic total occlusion, *PCI* percutaneous coronary intervention, *MI* myocardial infarction, *LVEF* left ventricular ejection fraction, *OR* odds ratio, *CI* confidential interval

**B. Death**

|  | **Univariable analysis** | | | **Multivariable analysis** | | |
| --- | --- | --- | --- | --- | --- | --- |
|  | **OR** | **95%CI** | **p Value** | **OR** | **95%CI** | **p Value** |
| **eGFR** | 0.972 | 0.946-0.999 | 0.042 | 0.972 | 0.946-0.999 | 0.042 |

*CTO* chronic total occlusion, *PCI* percutaneous coronary intervention, *eGFR* estimated glomerular filtration rate, *OR* odds ratio, *CI* confidential interval

**Additional file 1: Table S4 Univariable Logistic Regression for 1-year Clinical Outcomes in Diabetic Patients with Successful CTO-PCI**


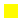


**A. MACE**

|  | **OR** | **95%CI** | **p Value** |
| --- | --- | --- | --- |
| **Age** | 1.033 | 1.003-1.064 | 0.031 |
| **CrCL** | 0.990 | 0.980-1.000 | 0.047 |
| **LVEF≤35%** | 3.863 | 1.812-8.235 | <0.01 |
| **Number of lesions per patient** | 1.680 | 1.157-2.439 | 0.006 |
| **Tortuosity≥45°** | 1.917 | 1.031-3.564 | 0.040 |

*CTO* chronic total occlusion, *PCI* percutaneous coronary intervention, *CrCL* creatinine clearance, *LVEF* left ventricular ejection fraction, *OR* odds ratio, *CI* confidential interval


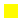


**B. Death**

|  | **OR** | **95%CI** | **p Value** |
| --- | --- | --- | --- |
| **Age** | 1.058 | 1.009-1.109 | 0.019 |
| **Males** | 0.320 | 0.108-0.950 | 0.040 |
| **Scr** | 1.006 | 1.001-1.011 | 0.016 |
| **eGFR** | 0.979 | 0.962-0.997 | 0.024 |
| **CrCL** | 0.980 | 0.964-0.997 | 0.019 |
| **LVEF≤35%** | 3.709 | 1.323-10.398 | 0.013 |
| **LM lesion** | 3.065 | 1.213-7.741 | 0.018 |
| **Number of lesions per patient** | 2.308 | 1.233-4.320 | 0.009 |
| **Contrast volume** | 0.995 | 0.991-0.999 | 0.024 |

*CTO* chronic total occlusion, *PCI* percutaneous coronary intervention, *Scr* serum creatinine, *eGFR* estimated glomerular filtration rate, *CrCL* creatinine clearance, *LVEF* left ventricular ejection fraction, *LM* left main coronary artery, *OR* odds ratio, *CI* confidential interval
